# Supercritical Fluid Extraction of Bacterial and Archaeal Lipid Biomarkers from Anaerobically Digested Sludge

**DOI:** 10.3390/ijms13033022

**Published:** 2012-03-06

**Authors:** Muhammad Hanif, Yoichi Atsuta, Koichi Fujie, Hiroyuki Daimon

**Affiliations:** 1 Department of Environmental and Life Sciences, Toyohashi University of Technology, Aichi 441-8580, Japan; E-Mails: hanif@water.ens.tut.ac.jp (M.H.); atsuta@water.ens.tut.ac.jp (Y.A.); 2 Center for Energy Resources Development, Agency for the Assessment and Application of Technology, Jakarta 10340, Indonesia; 3 Graduate School of Environment and Information Sciences, Yokohama National University, Kanagawa 240-8501, Japan; E-Mail: fujie@ynu.ac.jp

**Keywords:** anaerobically digested sludge, multiple response optimization, phospholipid fatty acid, phospholipid ether lipid, respiratory quinone, supercritical fluid extraction

## Abstract

Supercritical fluid extraction (SFE) was used in the analysis of bacterial respiratory quinone (RQ), bacterial phospholipid fatty acid (PLFA), and archaeal phospholipid ether lipid (PLEL) from anaerobically digested sludge. Bacterial RQ were determined using ultra performance liquid chromatography (UPLC). Determination of bacterial PLFA and archaeal PLEL was simultaneously performed using gas chromatography-mass spectrometry (GC-MS). The effects of pressure, temperature, and modifier concentration on the total amounts of RQ, PLFA, and PLEL were investigated by 23 experiments with five settings chosen for each variable. The optimal extraction conditions that were obtained through a multiple-response optimization included a pressure of 23.6 MPa, temperature of 77.6 °C, and 10.6% (v/v) of methanol as the modifier. Thirty nine components of microbial lipid biomarkers were identified in the anaerobically digested sludge. Overall, the SFE method proved to be more effective, rapid, and quantitative for simultaneously extracting bacterial and archaeal lipid biomarkers, compared to conventional organic solvent extraction. This work shows the potential application of SFE as a routine method for the comprehensive analysis of microbial community structures in environmental assessments using the lipid biomarkers profile.

## 1. Introduction

The anaerobic digestion process is increasingly recognized as an attractive alternative technology for the treatment of organic waste and wastewater [1]. Fundamental improvements in the anaerobic digestion process require a comprehensive understanding of the microbial community structure [2,3]. Therefore, current studies have focused on the development of a rapid and reliable method for analyzing the microbial community structure in the anaerobic digestion process.

A number of the microbial community analysis methods extract the cellular components of microorganisms directly from the sample without cultivation [4,5]. One of the most commonly used methods is the microbial lipid biomarkers profiling, including respiratory quinones (RQ), phospholipid fatty acids (PLFA), and phospholipid ether lipids (PLEL). These biomarkers have been established for the determination of bacterial and archaeal community structures in environmental samples [6]. A combination of the profiles of different lipid biomarkers could provide a comprehensive understanding of microbial interactions in complex communities [7].

RQ have been used as sensitive biomarkers for aerobic and anaerobic bacterial metabolism in environmental samples. They are classified into two major groups, ubiquinones and menaquinones. Ubiquinones and menaquinones occur most frequently in bacteria, and are in the bacterial plasma membrane where they function in electron transport [8–10]. PLFA have been utilized to determine the presence and abundance of specific microbial groups. PLFA are quickly degraded upon microbial death. They represent a fingerprint of the viable microbial community and do not function as storage compounds [11,12]. PLEL have been applied as biomarkers for archaea in an anaerobic digestion process. Archaea are characterized by unique diether and tetraether lipids, which are not found in other organisms [13].

The most widely used extraction method for the analysis of microbial lipid biomarkers is the Bligh and Dyer extraction with a chloroform-methanol mixture [14]. The samples are first extracted by the addition of this solution, and the lipid extracts are separated using acidic silicic column chromatography into neutral lipids, glycolipids and phospholipids. RQ from the neutral lipid fraction are quantified using high performance liquid chromatography (HPLC) [8,9] or atmospheric pressure chemical ionization and photoionization (APCI-PI) [15]. PLFA and PLEL from the phospholipid fraction are derivatized into their less polar ester- and ether-linked derivatives for gas chromatography analysis [4]. Another approach to extract individual PLEL uses an acidified chloroform-methanol solution [16]. Although these methods disrupt the cell sample [17], they use large quantities of hazardous solvents and are time-consuming.

Application of supercritical fluid extraction (SFE) to extract microbial lipids could lead to greater extraction efficiencies [17–19]. SFE also offers a faster extraction and uses less solvent [20]. Carbon dioxide is the preferred extraction fluid in SFE because it has relatively low critical values (31.1 °C and 7.4 MPa). It is non-toxic and does not create environmental problems when used at the analytical scale.

Supercritical carbon dioxide (scCO_2_) extraction has previously demonstrated its efficiency in the analysis of microbial RQ [21] and PLFA [22] in activated sludge. Characterization of lipid biomarkers profiles using a three-step extraction system, including ‘flash’ SFE, SFE, and enhanced solvent extraction (ESE) has been proposed elsewhere [23]. Combined scCO_2_ extraction and chemical derivatization has been used to extract fatty acids from pure and mixed bacterial cultures [24,25]. Supercritical fluid chromatography (SFC) has also been used to analyze pure archaeal cultures [26]. A few studies have reported the application of SFE in the analysis of microbial lipid biomarkers in environmental samples. In this study, a modified scCO_2_ was applied for the simultaneous extraction of microbial RQ, PLFA, and PLEL from anaerobically digested sludge.

Development of SFE method requires careful optimization of several parameters. The optimization of one SFE variable at a time using traditional trial-and-error method is a time-consuming process [27]. Therefore, a statistical experimental design was used in the present study to optimize the SFE conditions, and explore the relationships among several variables and one or more response variables.

The purpose of the present investigation was to study the applicability of scCO_2_ to simultaneously extract microbial RQ, PLFA, and PLEL in digested sludge from an anaerobic digestion process. The effect of pressure, temperature, and modifier concentration on the total amounts of RQ, PLFA, and PLEL extracted was investigated using a response surface methodology with a central composite design. Simultaneous multiple-response optimization was applied to achieve optimal operating conditions by maximizing all of the dependent variables. The conventional organic solvent extraction method was also used to evaluate the reliability of the SFE method. This work is one of the first investigations on the optimization of SFE for the simultaneous determination of bacterial and archaeal lipid biomarkers in an anaerobic digestion system using an experimental design procedure.

## 2. Results and Discussion

### 2.1. Model Fitting and Statistical Analysis

Preliminary experiments demonstrated that the pressure, temperature and modifier concentration were the main factors affecting the total amount of microbial lipids in the scCO_2_ extraction. The range of pressures, temperatures and modifier concentrations were defined by considering the deterioration of bacterial and archaeal cell with increasing temperature [21,22], and the alteration of cell structure with increasing pressure [19]. Therefore, the effects of pressure (10–30 MPa), temperature (60–100 °C) and modifier concentration (5–15%, v/v) on the total amounts of RQ, PLFA, and PLEL extracted were investigated. The central composite design matrix of the SFE method and total amounts of the microbial lipids that were extracted from anaerobically digested sludge are reported in Table 1. The independent and dependent variables were analyzed to obtain regression equations that could estimate the total amounts of lipid biomarkers extracted within a given range.

Table 2 shows the regressions coefficients that were obtained by fitting the experimental data to the second-order response models for the total amounts of RQ, PLFA, and PLEL extracted. The goodness of the fit of models was verified by the coefficient of determination (*R*^2^). In this experiment, the *R*^2^ values for RQ, PLFA, and PLEL were 0.86, 0.90 and 0.88, respectively. The experimental data were in agreement with the predicted values for the total amounts of RQ, PLFA, and PLEL extracted. The values of adjusted *R*^2^ ranged from 0.76 to 0.83, suggesting that the total variation in the total amounts of RQ, PLFA, and PLEL was attributed to the independent variables. In addition, the portion of total variation that was not explained by the models ranged from 0.17% to 0.24%.

**Figure 1:**
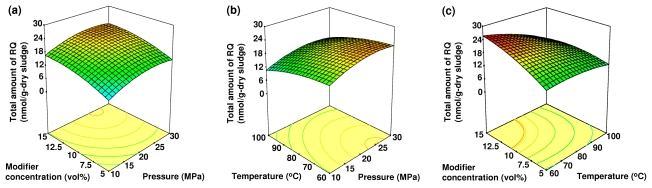
The 3-D response surface plot of the effects of the following independent variables on the total amount of RQ extracted: (**a**) the modifier concentration and pressure at a constant temperature (80 °C); (**b**) the temperature and pressure at constant modifier concentration (10%, v/v); and (**c**) the modifier concentration and temperature at constant pressure (20 MPa).

**Figure 2:**
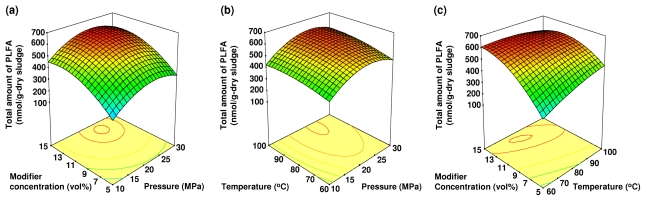
The 3-D response surface plot of the effects of the following independent variables on the total amount of PLFA extracted: (**a**) the modifier concentration and pressure at a constant temperature (80 °C); (**b**) the temperature and pressure at constant modifier concentration (10%, v/v); and (**c**) the modifier concentration and temperature at constant pressure (20 MPa).

**Figure 3:**
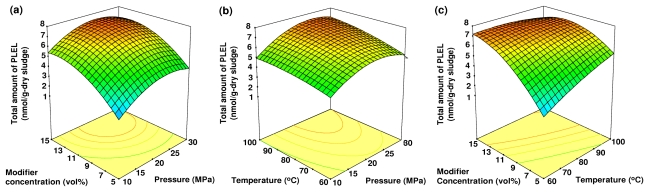
The 3-D response surface plot of the effects of the following independent variables on the total amount of PLEL extracted: (**a**) the modifier concentration and pressure at a constant temperature (80 °C); (**b**) the temperature and pressure at constant modifier concentration (10%, v/v); and (**c**) the modifier concentration and temperature at constant pressure (20 MPa).

Analysis of variance (ANOVA) was applied to evaluate the significance of different model equations associated with models parameters. A summary of the ANOVA for each microbial lipid biomarker from anaerobically digested sludge is shown in Table 3. The ANOVA of the quadratic regression model from the experimental data of microbial lipid biomarkers demonstrated that the model was highly significant with Fisher’s *F*-test values ranged from 8.84 to 12.63 (*p*-value < 0.01). The lack of fit *F*-value ranged from 1.05 to 2.43, and implied that the lack of fit was not significant relative to the pure error. A visualization of the interactions between two experimental factors with the third factor, which was held constant at its central level, is plotted by the three-dimensional (3-D) response surface in Figures 1–3.

### 2.2. The Effects of Pressure, Temperature and Modifier Concentration on the Total Amount of RQ

Figure 1a–c shows the interactions and effects of pressure, temperature, and modifier (methanol) concentration on the total amount of RQ extracted. The total amount of RQ extracted increased with increasing modifier concentration. However, increasing the extraction temperature resulted in a decrease in the total amount of RQ extracted in the tested conditions. All independent variables significantly affected the total amount of RQ extracted, with temperature exhibiting a negative influence. The interaction between the temperature and modifier concentration had a significant effect on the total amount of RQ extracted (*p*-value < 0.01, Table 2). Pressure increased the total amount of RQ extracted up to approximately 22 MPa, with no significantly further improvement thereafter. When considering the variables individually, the extraction conditions that maximize the total amount of RQ extracted were 22 MPa, 60 °C and a 15% (v/v) modifier concentration.

Increasing temperature is known to reduce the solvent density and consequently decrease the total amount of RQ extracted at constant pressure. At the same time, higher temperatures promote the solubility of the solute and increase the yield by high mass transfer of solute in the matrix and/or from the matrix to the solvent [28]. Therefore, the increase in temperature could have either a positive or a negative effect based on the balance between CO_2_ density and solute vapor pressure. An increase in pressure results in an increase in CO_2_ density by increasing the solvating power of the supercritical fluid [20]. An increase in pressure from 10 to 20 MPa induced a steady increase in the total amount of RQ extracted in this study.

### 2.3. The Effects of Pressure, Temperature and Modifier Concentration on the Total Amount of PLFA

The effects of pressure, temperature and modifier concentration on the total amount of PLFA extracted from anaerobically digested sludge are shown in Figure 2a–c. All independent variables significantly affected the total amount of PLFA extracted (*p*-value < 0.01, Table 2). The complex interaction between the temperature and modifier concentration had a negative effect. At a fixed extraction pressure of 20 MPa, the total amount of PLFA increased with increasing modifier concentration and temperature. It reached a maximum value at approximately 80 °C and 13% (v/v) of modifier concentration with no further improvement thereafter.

In this study, the total amount of PLFA extracted was increased with increasing pressure from 10 to 25 MPa. Above this range of pressure, a decrease in the total amount of PLFA extracted with increasing pressure was observed. The volatility and polarity of the extracted compounds might be responsible for these results and we can conclude that the composition of the extract can be controlled by pressure. When considering the variables individually, the extraction conditions that maximize the total amount of PLFA extracted were 25 MPa, 80 °C and a 13% (v/v) modifier concentration. These results indicate that the extraction of PLFA requires a higher temperature compared to that of RQ.

### 2.4. The Effects of Pressure, Temperature and Modifier Concentration on the Total Amount of PLEL

The effects of pressure, temperature and modifier concentration on the total amount of PLEL extracted from anaerobically digested sludge are shown in Figure 3a–c. Although the total amount of RQ extracted decreased with temperature, the total amount of PLEL exhibited the opposite trend. The modifier concentration showed the most significant effect (*p*-value < 0.01, Table 2).

At a given modifier concentration, the total amount of PLEL extracted increased rapidly with increasing extraction temperature. The total amount of PLEL extracted increased with increasing modifier concentration and reached a maximum value at a given pressure. With further increase in modifier concentration, the total amount of PLEL extracted slightly decreased. The fluid density is highly sensitive to temperature, especially near the system’s critical pressure. Therefore, the total amounts of PLEL extracted changed over the range from 60 to 100 °C. A moderate increase in temperature leads to a large decrease in fluid density, with a consequent reduction in solubility [29]. However, an increase in temperature will also accelerate mass transfer and improve the total amount of extracted compounds. Extraction conditions that maximized the total amount of PLEL extracted were 27 MPa, 95 °C and a 15% (v/v) modifier concentration.

### 2.5. Multiple-Response Optimization

A multiple-response optimization simultaneously maximizes all of the applied responses as described elsewhere [30]. In this study, the multiple-response optimization was performed by maximizing all of the dependent variables (total amounts of RQ, PLFA and PLEL extracted) together. The optimized extraction conditions were a pressure of 23.6 MPa, temperature of 77.6 °C, and 10.6% (v/v) of modifier concentration. The total amounts of RQ, PLFA, and PLEL were calculated to be 21.27, 596.11, and 6.98 nmol/g-dry sludge, respectively, under these conditions.

### 2.6. Comparison of Supercritical CO_2_ Extraction and Organic Solvent Extraction

The optimized extraction conditions from the previous experiments were applied to the scCO_2_ extraction experiment. Verification experiments were performed five times under the optimized conditions (23.6 MPa, 77.6 °C, and 10.6% (v/v) methanol). The total amounts of RQ, PLFA, and PLEL obtained were 22.17, 604.61, and 6.78 nmol/g-dry sludge, respectively, with relative standard deviations (RSDs) of 9.02%, 11.04%, and 3.68%, respectively.

**Figure 4:**
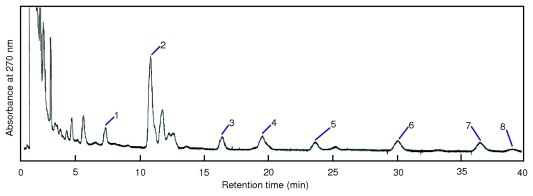
Ultra performance liquid chromatography (UPLC) chromatograms of RQ extracted from anaerobically digested sludge under the optimized scCO_2_ extraction conditions. The numbers near the peaks are as follows: (1) MK-6, (2) MK-7, (3) MK-8, (4) MK-8(H_2_), (5) MK-9, (6) MK-9(H_2_), (7) MK-9(H_4_), and (8) MK-10.

**Figure 5:**
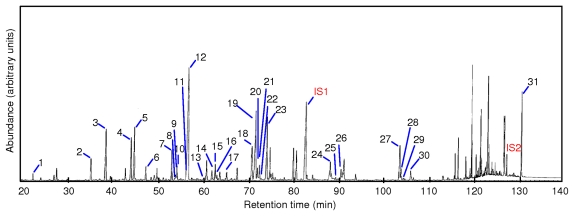
GC-MS chromatograms of PLFA and PLEL extracted from anaerobically digested sludge under optimal scCO_2_ extraction conditions. The numbers near the peaks are as follows: (1) 12:0, (2) i14:0, (3) 14:0, (4) i15:0, (5) a15:0, (6) 15:0, (7) i16:0, (8) 16:1*ω*9c, (9) 16:1*ω*8c, (10) 16:1*ω*7c, (11) 16:1*ω*5c, (12) 16:0, (13) 10Me16:0, (14) i17:0, (15) a17:0, (16) cy17:0, (17) 17:0, (18) 18:2, (19) 18:1*ω*9c, (20) 18:1*ω*7c, (21) 18:1*ω*9t, (22) 18:1*ω*8c, (23) 18:0, (IS1) the internal standard nonadecanoic acid methyl ester, 19:0, (24) 20:1*ω*9c, (25) 20:1*ω*9t (26) 20:0 (27) 22:1*ω*9c, (28) 22:1*ω*9t, (29) 22:1*ω*7c, (30) 22:0, (IS2) the internal standard 1,2-di-*O*-hexadecyl-rac-glycerol, and (31) diether(DE)-lipid.

The ultra performance liquid chromatography (UPLC) and GC-MS chromatograms of the microbial lipid biomarkers extracted from the anaerobically digested sludge by SFE are shown in Figures 4 and 5. RQ were determined by UPLC, while PLFA and PLEL were simultaneously determined by GC-MS. Eight types of RQ, 30 types of PLFA, and 1 type of PLEL were identified in the samples, illustrating the sensitivity of SFE technique for the simultaneous extraction of both bacterial and archaeal lipids from anaerobically digested sludge. The same numbers and types of microbial lipid biomarkers were obtained using the organic solvent extraction method.

**Figure 6:**
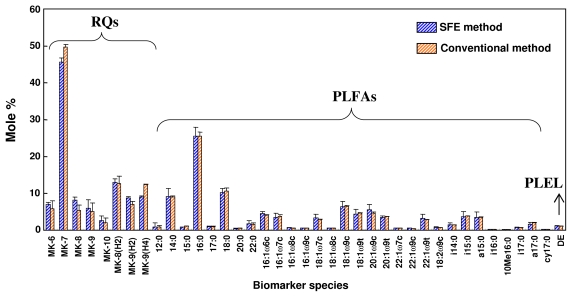
The mole percentages of microbial RQ, PLFA and PLEL extracted from anaerobically digested sludge by the SFE and conventional organic solvent extraction methods.

Figure 6 shows the mole concentrations of microbial RQ, PLFA, and PLEL extracted from anaerobically digested sludge by SFE and conventional organic solvent extraction. A mole percent basis was used for RQ, PLFA, and PLEL data. It indicated the SFE method did not alter the microbial community structure information. The mole concentrations of the individual biomarkers were nearly identical for the two methods. Microbial lipid extracts from the SFE method were in agreement with the conventional solvent extraction method. However, SFE method proved to be simple, fast, and with less consumption of organic solvents.

The nomenclature of the PLFA used in this study is designated by the total number of carbon atoms, the number of double bonds, and the position of the double bond closest to the methyl end (*ω*) of the molecule [31]. For example, the PLFA 18:2*ω*6c is 18 carbons long, with two double bonds positioned six carbons from the methyl end of PLFA, and exhibit a *cis* configuration. The prefixes *i*, *a*, and *cy* indicate iso branching, anteiso branching and cyclopropyl groups, respectively. For RQ nomenclature, ubiquinones and menaquinones with *n* isoprene units in their side chain were abbreviated as UQ-*n* and MK-*n*, respectively. For example, UQ-8 indicates a ubiquinone with 8 isoprene units and MK-8(H_2_) denotes a menaquinone with 8 isoprene units and one double bond saturated with 2 hydrogen atoms [32]. The PLEL are classified into di- and bidiphytanylglycerol ether phospholipids. Di-*O*-phytanylglycerol ether or diether(DE)-lipid contains two 20 carbon saturated and tetra-*O*-dibiphytanylglycerol ether or tetraether(TE)-lipid contains two 40 carbon isoprenoid hydrocarbons in ether linkage to glycerols [33].

Microbial lipids were grouped into saturated fatty acids, gram-positive and gram-negative bacteria, actinobacteria, fungi, and methanogenic archaea according to the guidelines that have been described elsewhere [34–36]. Saturated fatty acid group consisted of 12:0, 14:0, 15:0, 16:0, 17:0, 18:0, 20:0 and 22:0 PLFA. Gram-positive bacteria group consisted of MK-6, MK-7, and MK-8 RQ, and i14:0, i15:0, a15:0, i16:0, i17:0 and a17:0 PLFA. Gram-negative bacteria consisted of 16:1*ω*7c, 16:1*ω*8c, 16:1*ω*9c, 18:1*ω*7c, 18:1*ω*8c and cy17:0 PLFA. Actinobacteria group consisted of MK-9, MK-10, MK-8(H_2_), MK-9(H_2_), and MK-9(H_4_) RQ and 10Me16:0 PLFA. Fungi group consisted of 16:1*ω*5c, 18:1*ω*9c, 18:1*ω*9t, 18:2*ω*6c, 20:1*ω*9c, 20:1*ω*9t, 22:1*ω*7c, 22:1*ω*9c and 22:1*ω*9t PLFA. Methanogenic archaea group consisted of diether(DE)-lipid derived from PLEL.

## 3. Experimental Section

### 3.1. Chemicals and Standards

All solvents were HPLC grade, and all other chemicals were analytical grade. Methanol, acetone, chloroform, ethanol and hexane were purchased from Wako Co., Japan. The fatty acid standard (nonadecanoic acid methyl ester), diether lipid standard (1,2-di-*O*-hexadecyl-rac-glycerol), bis(trimethylsilyl)trifluoroacetamide (BSTFA), and 3 N methanolic-HCl were purchased from Sigma-Aldrich Co., Japan. The ubiquinone (UQ-10) and menaquinone (MK-7) standards were purchased from Nacalai Tesque Co., Japan. Standard stock solutions were stored at 4 °C. Sep-Pak^®^ Plus Silica cartridges (Cat. no. WAT020520) were purchased from Waters Co., Japan.

### 3.2. Sample Preparation

The anaerobically digested sludge used in this study was obtained from an 8-L lab-scale semi-automatic continuously stirred bioreactor (38 °C) treating a mixture of cow manure with food waste at the Department of Environmental and Life Sciences, Toyohashi University of Technology, Japan. Anaerobically digested sludge is a semi-solid material that is released during anaerobic digestion as a complex mixture of microbial fragments and organic colloids [37]. Prior to SFE extraction, the digested sludge samples were dried in a vacuum-freeze dryer for 24 h and homogenized by crushing. Freeze-dried digested sludge samples were stored at −20 °C until analysis.

### 3.3. Organic Solvent Extraction

Samples were extracted according to the modified Bligh and Dyer method [4] using a mixture of chloroform, methanol and phosphate buffer (50 mM, pH 7.4) at a ratio of 1:2:0.8 (v/v/v). After overnight extraction, chloroform and milli-pure water were added to the extract in equal amounts to yield a two-phase system with a final volume of 1:1:0.9 (v/v/v). The supernatant (upper phase) was transferred with a pipette into a test tube. The solvent was removed from the test tube under constant nitrogen flow with a water bath at a temperature that was not higher than 37 °C. The total lipid extract was fractionated into neutral lipids, glycolipids, and phospholipids, with chloroform (10 mL), acetone (10 mL) and methanol (10 mL), on acidic silicic columns. The neutral lipid fraction was cleaned with 0.2 μm spin filter. The extracts containing RQ were resolved in 200 μL acetone, and quantified by UPLC [16]. The phospholipid fraction was derivatized to form PLFA as theirs fatty acid methyl ester and PLEL as theirs etherlipid derivatives. PLFA and PLEL were simultaneously quantified using GC-MS [33,38]. The organic solvent extraction protocol for this study took 3 days to complete.

### 3.4. Supercritical Fluid Extraction

All supercritical fluid extractions were performed using an in-house constructed supercritical fluid extraction system. This system is equipped with a high-pressure pump (SCF-201, Jasco Co., Japan), a backpressure regulator (880-81, Jasco Co., Japan) and an oven (GC A353, GL Sciences Inc., Japan). In total, 100 mg of freeze-dried digested sludge was placed in a 1-mL stainless steel extraction cell. One end of the extraction cell was connected to a high-pressure pump through a 2-m preheating coil (0.5 mm i.d. stainless steel tubing), which was placed in an oven. The liquid CO_2_ was compressed with a cooler and then allowed to flow into the extraction vessel at a flow rate of 2.7 mL/min. The liquid CO_2_ and modifier (methanol) were continuously mixed in-line. The modifier is a polar or non-polar miscible solvent, and it is used to modify the polarity and solvent strength of the SFE. All operations were performed in dynamic extraction mode for 15 min. The dynamic mode means the mixture was passed over the activated sludge sample inside the extraction vessel. During the extraction, extracted lipids were collected in a dark glass tube to prevent photodecomposition and then dried under a stream of nitrogen gas.

### 3.5. Lipid Fractionation and Derivatization Procedures

The total lipid extracted obtained from SFE was fractionated into neutral lipids, glycolipids and phospholipids with chloroform (5 mL), acetone (5 mL) and methanol (5 mL) on SepPak^®^ Plus silica cartridges. The neutral lipid fraction was collected in a 15-mL glass test tube and evaporated to dryness under a stream of nitrogen. The neutral lipid fraction containing ubiquinones and menaquinones were resolved in 100 μL acetone and analyzed using UPLC without further purification [15]. The fraction containing phospholipids was also collected in a 15-mL glass test tube and evaporated to dryness under a stream of nitrogen.

Prior to GC-MS analysis, the PLFA in phospholipid fraction were derivatized to produce their fatty acid methyl esters (FAMEs), as described elsewhere [38]. Phospholipid extracts were incubated at 80 °C in a heated block for 1 h after the addition of a methanolic HCl solution. The tubes were vortex-mixed every 10 min during the incubation and subsequently cooled for approximately 20 min. The phospholipid extracts were vortex-mixed for 15 s in 3 mL of hexane and centrifuged for 5 min at 2000 rpm. The hexane phase containing the FAMEs was evaporated to dryness under nitrogen. Diether lipids were converted to their corresponding *O*-trimethylsilylethers by treatment with bis(trimetylsilyl)trifluoroacetamide (BSTFA) [33]. An internal standard mixture of nonadecanoic acid methyl ester and 1,2-di-*O*-hexadecyl-rac-glycerol was added to the final phase containing FAMEs and etherlipids. The final phase was then transferred to an amber vial and stored at −20 °C.

### 3.6. UPLC Analysis of RQ Extracted from Anaerobically Digested Sludge

Chromatographic separation of RQ was performed using the Waters Acquity UPLC™ system (Milford, MA, USA), including a binary solvent delivery manager, a sample manager, a column compartment and a photo-diode array detector (PDA-2996, Waters Co., USA), connected with Waters Empower Software. The analytical column was a Water Acquity UPLC™ BEH C_18_ column (50 mm × 2.1 mm, 1.7 μm particle size). The mobile phase consisted of 100% methanol and pumped at a flow rate of 0.5 mL/min. Chromatography was performed at 35 ± 1 °C with a run time of 40 min. The autosampler temperature was set at 4.0 ± 1 °C, and the sample injection volume was 10 μL. The detector wavelengths were set at 275 nm and 270 nm for ubiquinones and menaquinones, respectively [21].

### 3.7. Gas Chromatography Analysis of PLFA and PLEL Extracted from Anaerobically Digested Sludge

PLFA and PLEL concentrations were quantified on a Hewlett-Packard 6890 GC Series system with a 5973 N Mass Selective Detector (Wilmington, DE, USA). Standard GC analysis for the simultaneous determination of PLFA and PLEL has been described elsewhere [31]. In contrast, we used a cross-linked methyl silicone fused-silica HP-5MS capillary column (30 m × 0.25 mm i.d. × 0.25 μm film thickness), and a total run time of 138 min. Helium was used as the carrier gas at a flow rate of 0.9 mL/min. NIST 98 MS software library was used for PLFA and PLEL compounds identification. The initial oven temperature was 50 °C for 1 min and was increased at a rate of 30 °C/min (to reach 110 °C), 1 °C/min (to reach 220 °C), and 10 °C/min (to reach 320 °C). The final temperature was maintained for 15 min. One microliter of samples was injected splitless for 0.75 min at injector temperature of 320 °C. The temperature of the transfer line between GC and MS was 320 °C. Nonadecanoic acid methyl ester and 1,2-di-*O*-hexadecyl-rac-glycerol were used as internal standards.

### 3.8. Experimental Design

The selection of the optimum combination of input variables can be solved by the development of mathematical model. In this work, the experimental design consisted of a response surface methodology with a central composite design (CCD) with three variables and five settings. Preliminary experiments indicated that the variables, such as pressure, temperature and methanol concentration, were the main factors that affected the total amount of lipid biomarkers extracted. Thus, these parameters were selected as the independent variables, and output data (total amounts of RQ, PLFA and PLEL) as the dependent variables. The selected experimental factors in coded and actual settings for the SFE are shown in Table 1. The upper limit of a factor was coded as +1.68 and lower limit as −1.68. The intermediate value was coded as 0. Factorial points were coded as +1 and −1. In the CCD test, 14 factorial points and 9 central points were employed to fit the full quadratic model. These runs and their combination sets are called experimental design matrix [39]. Nine replicates at the center of the design were used to estimate a pure error sum of the square. The experiments were conducted in a random order to maximize the effects of unexplained variability in the observed responses. Thus, 23 runs were carried out to estimate the effect of SFE parameters.

The following second-order polynomial equation was used:

(1) $$Y=\beta_{0}+\sum_{j=1}^{k}\beta_{j}x_{j}+\sum_{j=1}^{k}\beta_{jj}x_{j}^{2}+\sum \sum_{i<j}\beta_{ij}x_{i}x_{j}\;\;\;(k=3)$$

This equation used the following variables: *Y* is the predicted response; *k* is the number of factor variables; *β*_0_ is the model constant; *β_j_* is the linear coefficient; *β_jj_* is the quadratic coefficient; *β_ij_* is the interaction coefficient; and *x_i_* and *x_j_* are the independent coded variables. Design Expert^®^ software (v8 trial, Stat-Ease Inc., Minneapolis, MN, USA) was used for the regression analysis and visualization of the response surface data. The fit of the regression model was checked by the adjusted coefficient of determination (*R*^2^). The statistical significance of the adjusted model was determined by the application of Fisher’s *F*-test. Analysis of variance (ANOVA) was performed to evaluate significant differences between independent variables. The adequacy of the model was determined by evaluating the pure error, the lack of fit, the coefficient of determination (*p*-value) and the Fisher test value (*F*-value).

## 4. Conclusions

We investigated an alternative method for the extraction of microbial lipid biomarkers from anaerobically digested sludge using scCO_2_ extraction to replace the conventional organic solvent extraction method. Our optimized method simultaneously detected RQ, PLFA, and PLEL. The multiple-response optimization maximized all the dependent variables (total amounts of RQ, PLFA and PLEL extracted) together. Optimal extraction conditions were achieved at 23.6 MPa, 77.6 °C and 10.6% (v/v) methanol. Under these conditions, the total amounts of RQ, PLFA, and PLEL were 22.17, 604.61 and 6.78 nmol/g-dry sludge, respectively, with relative standard deviations (RSDs) of 9.02%, 11.04% and 3.68%, respectively. The experimental values agreed with predicted values, and the scCO_2_ extraction results were comparable with those obtained by conventional organic solvent extraction. Eight menaquinone components, 30 fatty acid components and 1 etherlipid component were identified in the samples, indicating the sensitivity of SFE method for the simultaneous extraction of both bacterial and archaeal lipids from anaerobically digested sludge. The SFE method has the potential to drastically reduce the amount of solvent used and extraction time needed, and could simplify the procedure. This method could be an effective technique for analyzing microbial lipid biomarkers in environmental samples, with the possibility of extended application and automation.

## Figures and Tables

**Table 1:** The central composite design matrix of the supercritical fluid extraction (SFE) method and the total amounts of the microbial respiratory quinone (RQ), phospholipid fatty acid (PLFA), and phospholipid ether lipid (PLEL) that were extracted from anaerobically digested sludge.

Design matrix					Total amounts b		

		Factors			Microbial lipid biomarkers extracted		
		
No.	Run a	Pressure (MPa)	Temperature (°C)	Modifier (methanol %, v/v)	RQ (nmol/g)	PLFA (nmol/g)	PLEL (nmol/g)
1	10	−1 (10)	−1 (60)	−1 (5)	12.32	152.88	1.21
2	7	+1 (30)	−1 (60)	−1 (5)	15.57	164.06	1.26
3	16	−1 (10)	+1 (100)	−1 (5)	10.46	350.56	3.56
4	23	+1 (30)	+1 (100)	−1 (5)	12.26	420.13	5.52
5	2	−1 (10)	−1 (60)	+1 (15)	22.93	470.31	5.57
6	12	+1 (30)	−1 (60)	+1 (15)	25.44	510.50	6.50
7	22	−1 (10)	+1 (100)	+1 (15)	10.34	459.38	5.72
8	19	+1 (30)	+1 (100)	+1 (15)	13.36	520.25	6.15
9	4	−1.68 (3.18)	0 (80)	0 (10)	4.97	112.14	0.89
10	18	+1.68 (36.82)	0 (80)	0 (10)	18.02	470.31	5.64
11	13	0 (20)	−1.68 (46.36)	0 (10)	18.38	487.81	4.86
12	8	0 (20)	+1.68 (113.64)	0 (10)	10.92	510.50	6.48
13	1	0 (20)	0 (80)	−1.68 (1.59)	6.48	125.32	0.56
14	9	0 (20)	0 (80)	+1.68 (18.41)	20.97	535.94	6.15
15	11	0 (20)	0 (80)	0 (10)	23.60	610.75	7.32
16	21	0 (20)	0 (80)	0 (10)	21.08	590.25	5.95
17	5	0 (20)	0 (80)	0 (10)	20.68	600.38	7.13
18	17	0 (20)	0 (80)	0 (10)	15.96	560.94	8.12
19	3	0 (20)	0 (80)	0 (10)	19.41	598.63	7.51
20	20	0 (20)	0 (80)	0 (10)	17.81	615.13	4.95
21	15	0 (20)	0 (80)	0 (10)	21.05	430.69	5.76
22	6	0 (20)	0 (80)	0 (10)	19.75	632.25	6.85
23	14	0 (20)	0 (80)	0 (10)	20.90	590.07	7.21

a Experiments were performed in random order;

b Mean of two individual determinations was expressed in nmol/g-dry sludge.

**Table 2:** The response surface quadratic polynomial model for the SFE of microbial RQ, PLFA, and PLEL from anaerobically digested sludge.

Factor	Coefficient Estimate a	RQ	PLFA	PLEL
Intercept	*β*_0_	19.97	580.58	6.74
*x*_1_ (pressure)	*β*_1_	2.38 **	57.42 **	0.82 *
*x*_2_ (temperature)	*β*_2_	−3.10 **	35.93 *	0.65 *
*x*_3_ (modifier)	*β*_3_	3.36 **	114.48 **	1.61 **
*x*_1_^2^ (pressure)	*β*_12_	−2.51 **	−56.87 **	−1.12 **
*x*_2_^2^ (temperature)	*β*_13_	−1.39	−98.32	−0.27
*x*_3_^2^ (modifier)	*β*_23_	−1.72 *	−84.39 **	−1.09 **
*x*_1_*x*_2_ (pressure × temperature)	*β*_11_	−0.12	9.88	0.20
*x*_1_*x*_3_ (pressure × modifier)	*β*_22_	0.06	2.54	−0.11
*x*_2_*x*_3_ (temperature × modifier)	*β*_33_	−2.44 *	−56.87 *	−0.88 *
	*R*^2^	0.86	0.90	0.88
	Adjusted *R*^2^	0.76	0.83	0.80
	Standard Deviation	2.71	69.06	1.01

a *Y* = β_0_ + β_1_*x*_1_ + β_2_*x*_2_ + β_3_*x*_3_ + β_11_*x*_1_^2^ + β_22_*x*_2_^2^ + β_33_*x*_3_^2^ + β_11_*x*_1_*x*_2_ + β_13_*x*_1_*x*_3_ + β_23_*x*_2_*x*_3_;

* Significant at *p*-value < 0.05;

** Significant at *p*-value < 0.01.

**Table 3:** The analysis of variance (ANOVA) of the model for SFE of microbial RQ, PLFA, and PLEL from anaerobically digested sludge.

Source	Degrees of freedom	Sum of squares	Mean square	*F*-value	*p*-value
*RQ*					
Model	9	585.95	65.11	8.84	0.0003 **
Residual	13	95.75	7.37		
Lack of fit	5	57.72	11.54	2.43	0.1269
Pure error	8	38.02	4.75		
Corrected total	22	681.70			
*PLFA*					
Model	9	5.42 × 10^5^	6.02 × 10^4^	12.63	<0.0001 **
Residual	13	6.20 × 10^4^	4.77 × 10^3^		
Lack of fit	5	3.34 × 10^4^	6.69 × 10^3^	1.88	0.2042
Pure error	8	2.85 × 10^4^	3.56 × 10^3^		
Corrected total	22	6.04 × 10^5^			
*PLEL*					
Model	9	96.84	10.76	10.57	0.0001**
Residual	13	13.24	1.02		
Lack of fit	5	5.23	1.05	1.05	0.4536
Pure error	8	8.01	1.00		
Corrected total	22	110.08			

** Significant at *p*-value < 0.01.
